# Harmonising Depression and Anxiety Measures Across International Cohorts: A Framework Validated Using Measurement Invariance

**DOI:** 10.1002/mpr.70102

**Published:** 2026-08-27

**Authors:** James Lian, Pedro F. Zuccolo, Omid V. Ebrahimi, Daniel Fatori, Abhaya Adlakha, Juan F. De La Hoz, Younga H. Lee, Adriana Carneiro, Isabela M. Benseñor, Paulo A. Lotufo, Alessandra C. Goulart, Justin D. Tubbs, Devon Watts, Yu Zhou, Lorenza Dall’Aglio, Mihael Cudic, Morgane Kuenzi, CGMHC Consortium, Ronald C. Kessler, Vikram Patel, André Brunoni, Jordan W. Smoller, Sarah Bauermeister

**Affiliations:** ^1^ Department of Psychiatry Dementias Platform UK University of Oxford Warneford Hospital Oxford UK; ^2^ Departamento de Psiquiatria, Faculdade de Medicina FMUSP Universidade de Sao Paulo Sao Paulo Brazil; ^3^ Department of Experimental Psychology University of Oxford Oxford UK; ^4^ Department of Psychiatry University of Oxford Oxford UK; ^5^ Laboratorio de Psicopatologia e Terapeutica Psiquiatrica LIM‐23 Instituto de Psiquiatria Hospital das Clinicas HCFMUSP Faculdade de Medicina Universidade de Sao Paulo Sao Paulo Brazil; ^6^ Department of Psychiatry Massachusetts General Hospital Boston Massachusetts USA; ^7^ Centro de Pesquisa Clínica e Epidemiológica Hospital Universitário Universidade de São Paulo Sao Paulo Brazil; ^8^ Departamento de Epidemiologia, Faculdade de Saúde Pública Universidade de São Paulo Sao Paulo Brazil; ^9^ Department of Health Care Policy Harvard Medical School Boston Massachusetts USA; ^10^ Department of Global Health & Social Medicine Harvard Medical School Boston Massachusetts USA

**Keywords:** artificial intelligence, COVID‐19, data harmonization, longitudinal cohort study, multi‐group confirmatory factor analysis (MGCFA)

## Abstract

**Aims:**

Cross‐national research on mental health trajectories requires harmonised measures that are valid and comparable. However, measurement scales often differ between cohorts in item content and cultural context. This study aimed to develop a framework for harmonising mental health measures across international cohorts, focussing on depression and anxiety symptoms.

**Methods:**

We applied a structured, theory‐driven approach to align item content across two longitudinal ageing cohorts that used different self‐report instruments with heterogeneous item codings: ELSA‐UK and ELSA‐Brasil. Data were collected before, during, and after the COVID‐19 pandemic, providing a natural experiment for examining temporal changes in mental health. The harmonisation process involved: (1) mapping items to DSM‐5 symptom domains via expert review; (2) transforming response formats into harmonised ordinal indicators; (3) leveraging the Harmony AI tool to identify semantically equivalent items; and (4) testing measurement invariance across waves and cohorts using multi‐group confirmatory factor analysis (MGCFA).

**Results:**

Scalar invariance was achieved for depression in both longitudinal and cross‐cohort models, enabling meaningful latent mean comparisons between cohorts. For anxiety, scalar invariance was supported within but not across cohorts, likely due to the limited number of conceptually matching items.

**Conclusion:**

The proposed harmonisation framework demonstrates the feasibility of robust cross‐cultural comparisons of mental health constructs. Its success depends on the conceptual alignment and quality of available items. This approach provides a methodological template for future harmonisation efforts in global health and psychiatric epidemiology.

## 1 Introduction

Internationally harmonised health data have become increasingly essential for addressing key global health research questions (O'Connor et al. 2022). In epidemiological research, meaningful comparisons across countries, time points, and populations rely on the availability of harmonised measures (McElroy, Wood et al. 2024; Black et al. 2024). Harmonisation enables pooled analyses, enhances statistical power, and facilitates generalisability of findings. It also expands the range of contextual and demographic variations, such as socioeconomic status or public health policies, by integrating diverse samples from multiple settings (Fortier and et al. 2017; Fortier et al. 2011; Bauermeister et al. 2023). However, in the field of mental health, where assessment tools often vary in content, structure, and cultural interpretation, creating comparable measures across studies remains a significant methodological challenge (Cheng et al. 2024).

Several initiatives have published guidelines to promote standardised harmonisation practices (Fortier and et al. 2017) and successful examples are emerging (Fortier and et al. 2017; Wey et al. 2021; Moltrecht et al. 2024; McElroy, Wood et al. 2024; Maelstrom Research, 2024). Despite this progress, harmonising mental health data across heterogeneous cohorts remains challenging. In many cases, harmonisation is restricted to studies using identical instruments, such as the Health and Retirement Study (HRS) International Family of Studies, which employs the Centre for Epidemiologic Studies Depression Scale (CES‐D) across member cohorts (J. Lee and et al. 2024). When cohorts rely on different instruments, harmonisation strategies typically include retrospective item mapping, variable recoding, or the application of item response theory (IRT) (Mc Elroy and et al. 2020; Kennedy et al. 2024). These strategies, while valuable, face limitations: they may depend heavily on subjective decisions about item equivalence, impose strong assumptions such as unidimensionality, or fail to evaluate whether constructs are measured equivalently across time and groups (Black et al. 2024; Putnick and Bornstein 2016).

To address these limitations, researchers have increasingly called for harmonisation frameworks that explicitly test measurement equivalence across diverse populations (Alavi et al. 2026). Multi‐group confirmatory factor analysis (MGCFA) offers such a framework, providing a rigorous psychometric method for assessing whether latent constructs are measured consistently across cohorts, cultures, and timepoints (King et al. 2023). MGCFA involves fitting a series of increasingly constrained models—configural, metric, and scalar invariance—to evaluate whether observed differences reflect genuine variation in underlying constructs rather than artefacts of measurement bias or cultural interpretation. As such, MGCFA offers a scalable and statistically principled foundation for global harmonisation of health constructs, particularly when studies differ in language, format, or item selection (Putnick and Bornstein 2016; Van De Schoot et al. 2015).

Although prior work has examined measurement invariance either longitudinally or cross‐sectionally, few harmonisation strategies address both dimensions simultaneously (Davidov et al. 2014). Without evidence that a measure functions consistently across time and across populations, observed differences may conflate true change with artefacts of measurement (Welzel et al. 2023). Furthermore, studies examining measurement invariance of mental health instruments across cultures have largely focussed on validating individual questionnaires rather than harmonising different instruments across cohorts. For example, cross‐cultural invariance has been investigated for the PHQ‐9 and the Perceived Stress Scale across eight low‐ and middle‐income countries (LMICs), demonstrating that while configural and metric invariance can often be established, scalar invariance is frequently more difficult to achieve because of cultural differences in symptom expression and item functioning (Murray et al. 2022; Katus et al. 2022). To our knowledge, no prior study has combined expert‐driven item mapping, data‐driven semantic matching, harmonisation of heterogeneous response formats, and formal invariance testing across both time and cohorts in a single methodological pipeline.

The COVID‐19 pandemic provides a unique natural experiment for testing harmonisation methods due to its global reach, profound psychological impact, and variability in public health responses. While extensive research has documented health effects during the pandemic (Santomauro et al. 2021; Cénat et al. 2022; Miao et al. 2023; Phiri et al. 2021; Leung et al. 2022; Robinson et al. 2022), relatively little is known about cross‐national differences in mental health trajectories during this period. Countries varied widely in the timing, duration, and stringency of containment policies (Hale and et al. 2020), contributing to variability in mental health responses. Vulnerable groups, such as women, young people, and individuals from socioeconomically disadvantaged backgrounds, were disproportionately affected (Almeida et al. 2020; Zuccolo and et al. 2022; Hawrilenko et al. 2021; Hawks 2023; Fatori et al. 2022; Y. H. Lee et al. 2022). Moreover, mental health trends fluctuated over time, often peaking during initial lockdowns and attenuating thereafter (Iob et al. 2020; Fancourt et al. 2021; Bu et al. 2023; Magnúsdóttir et al. 2022; Ebrahimi et al. 2022; Pierce et al. 2021; Batterham and et al. 2021). These unique circumstances provide an opportunity to test whether harmonisation methods can yield valid, comparable mental health indicators across countries with different instruments, contexts, and pandemic responses.

### 1.1 Objectives

As part of the COVID Global Mental Health Consortium (CGMHC), we aimed to develop and apply a systematic harmonisation framework to generate depression and anxiety measures that are comparable both longitudinally and cross‐culturally, despite differences in item content and response formats across instruments. Data came from two culturally and socioeconomically distinct cohorts: the English Longitudinal Study of Ageing (ELSA‐UK) (Steptoe et al. 2013) and the Brazilian Longitudinal Study of Ageing (ELSA‐Brasil) (Schmidt et al. 2015; Brunoni et al. 2023). Using these studies, we implemented a multi‐step process combining expert item mapping, AI‐based semantic similarity matching, and multi‐group confirmatory factor analysis (MGCFA) to evaluate measurement invariance across time and cohorts. Our objective was to test the feasibility of this framework by deriving harmonised latent factor scores for depression and anxiety, and to highlight both its potential and its limitations for future cross‐national health research.

## 2 Methods

### 2.1 Data Sources

#### 2.1.1 Participating Cohorts

This study draws on two longitudinal population‐based cohorts: the English Longitudinal Study of Ageing (ELSA‐UK), and the ELSA‐Brasil COVID‐19 Mental Health Cohort study. Despite their similar names, these studies differ substantially in measurement instruments, cultural context, and data collection procedures.

**FIGURE 1:**
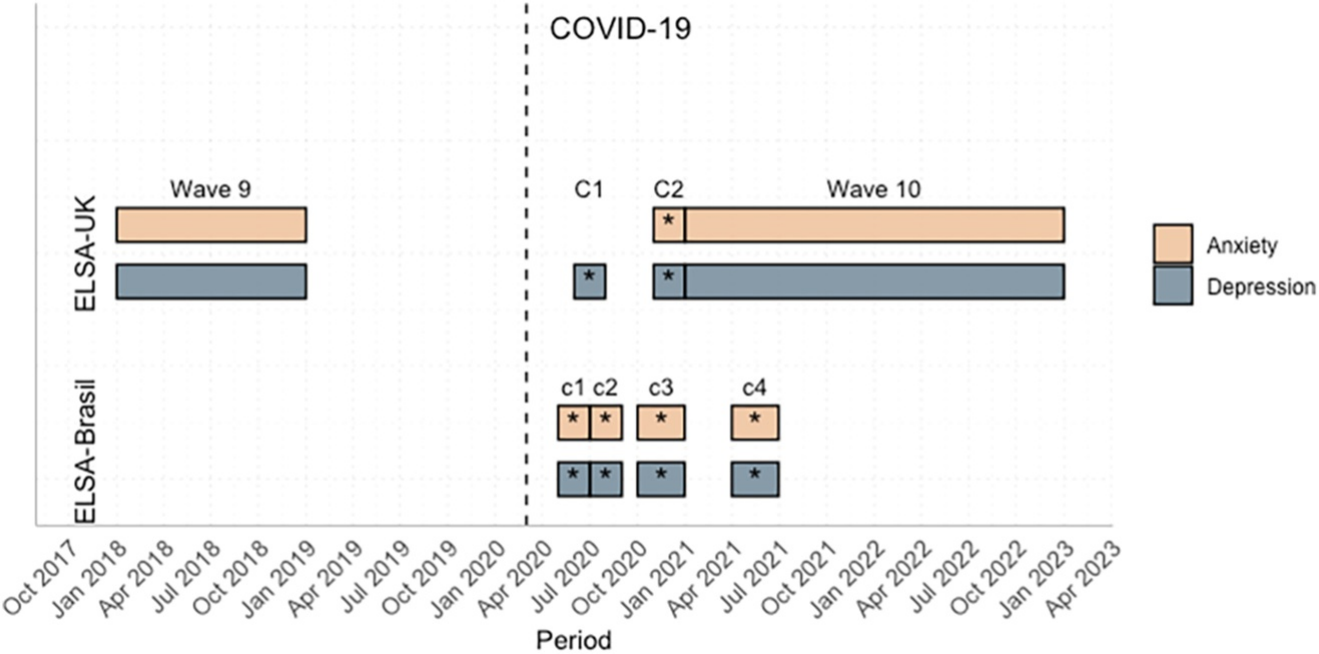
Timing of data collection around the COVID‐19 pandemic. ELSA‐UK waves (*N* = 11,004): Wave 9: 2018–2019, C1: June‐July 2020, C2: November, December 2020, and Wave 10: 2021–2023. ELSA‐Brasil waves (*N* = 2691): c1: May–July 2020, c2: July–September 2020, c3: October–December 2020, and c4: April to June 2021.

The ELSA‐UK cohort, launched in 2002, follows a nationally representative sample of adults aged 50 and older living in England, tracking health, social, and economic changes in later life. For this study, we used data from wave 9 (2018–2019), two COVID‐19 sub‐studies (C1: June–July 2020 and C2: November–December 2020), and wave 10 (2021–2023), comprising 13,695 participants. For both cohorts, we selected waves from 2018 to 2023 that were available in the DPUK portal and contained comparable mental health measures, rather than aligning assessments by calendar date. This ensured that harmonisation focussed on measurement equivalence, even though data collection periods differed between countries. Figure 1 depicts the timing of assessments included in these analyses. Ethical approval was obtained from relevant local committees for all waves and cohorts.

The ELSA‐Brasil cohort is a prospective study of 15,105 participants from six major Brazilian cities, established to investigate the clinical and sociodemographic determinants of chronic diseases and mortality in a middle‐income country context (Schmidt et al. 2015). Participants are university employees (active or retired), aged 35–74 years, and free from major neurocognitive disorders at enrolment. Nested within the larger study is ELSA Brasil COVID‐19. During the pandemic, participants from the São Paulo site (*n* = 4191) were invited to complete online assessments of mental health, performed in four waves: c1 (May–July 2020), c2 (July–September 2020), c3 (October–December 2020), and c4 (April–June 2021) (Brunoni et al. 2023; Fatori et al. 2022). A total of 2691 individuals who completed at least one wave were included in the analysis.

#### 2.1.2 Setting

All analyses were conducted within the secure Dementias Platform UK (DPUK) Data Portal (Dementias Platform UK 2024) using R version 4.1 between December 2024 and May 2025. Data were not downloaded or analysed externally.

### 2.2 Mental Health Measures

In ELSA‐Brasil, symptoms were measured using the Brazilian Portuguese version of the Depression, Anxiety and Stress Scale‐21 (DASS‐21), which had previously undergone cultural adaptation and psychometric validation for use in Brazil (Vignola and Tucci 2014). While all participant responses were collected using the validated Brazilian Portuguese instrument, for the purposes of harmonisation, item wording was translated into English by the research team to facilitate expert item mapping and semantic matching. Items were rated on a four‐point Likert scale (0–3) covering the past week, producing depression, anxiety, and stress subscales.

In ELSA‐UK, mental health was assessed with the CES‐D‐8 (O'Halloran et al. 2014), GAD‐7 (Spitzer et al. 2006), CASP‐12 (Wiggins et al. 2008), and ONS‐4 (Personal well‐being user guidance 2025). The CES‐D‐8 uses binary responses (yes/no), while the GAD‐7 includes seven four‐point items (0–3). CASP‐12 assesses quality of life on a four‐point scale, and ONS‐4 uses 0–10 ratings for life satisfaction, purpose, happiness, and anxiety.

### 2.3 Harmonisation Pipeline

**FIGURE 2:**
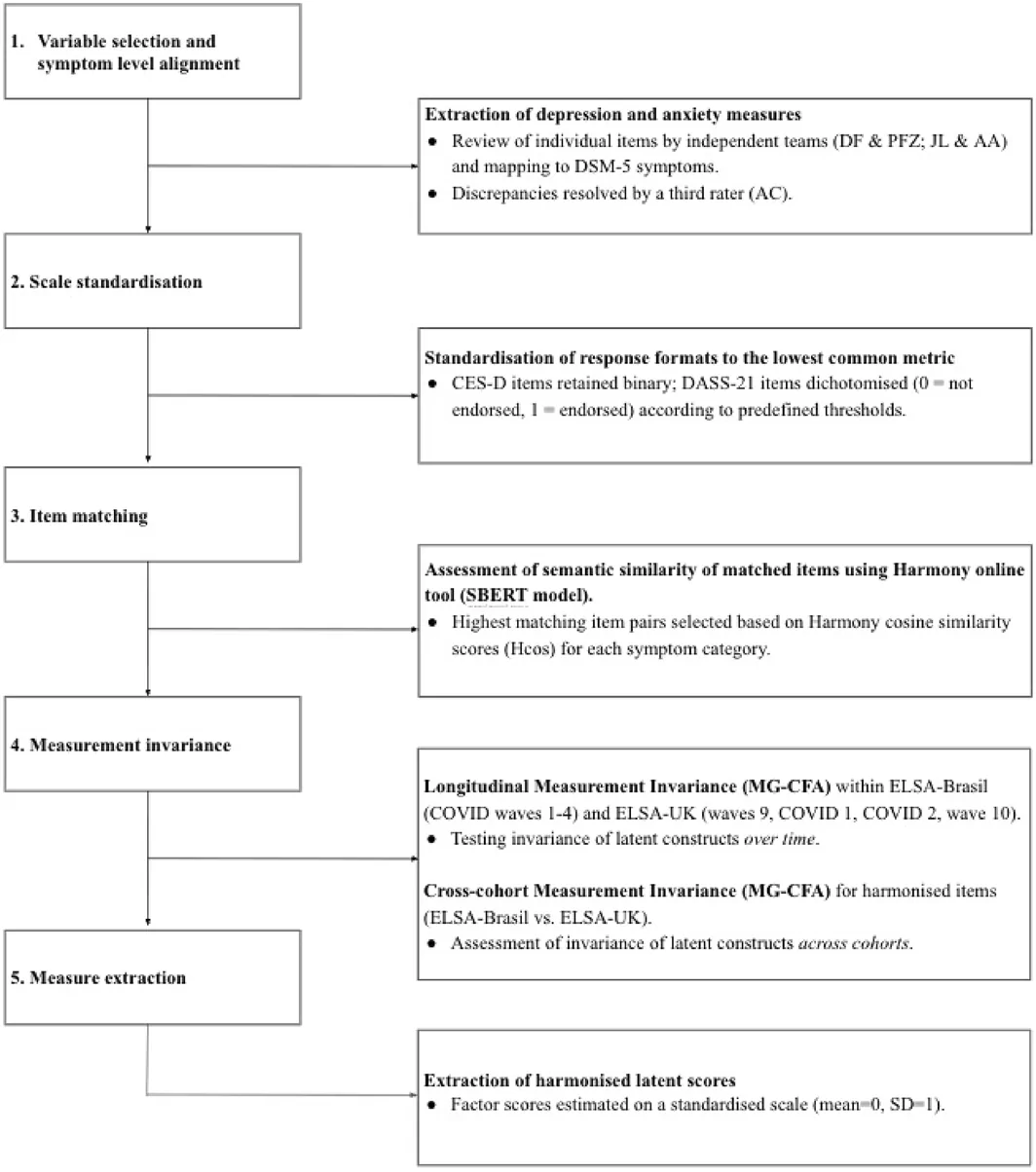
Harmonisation pipeline.

To facilitate cross‐cohort comparisons, a team of subject matter experts (PFZ, JL, DF, and AA) conducted a 5‐step process of retrospective harmonisation of mental health measures, as shown in Figure 2. The experts are either clinical psychologists and/or hold doctorates in psychiatric epidemiology.

#### 2.3.1 Variable Selection and Symptom Level Alignment

Data dictionaries from each cohort were reviewed to identify and extract all measures assessing depression and anxiety. Symptom categories for these conditions were defined based on DSM‐5‐TR criteria for major depressive disorder and generalised anxiety disorder. All relevant items were then compiled in a spreadsheet to allow for symptom‐level and item‐level alignment (see Supporting Information S1).

Two independent teams (DF and PFZ; JL and AA) mapped items to specific symptom domains (e.g., fatigue, worry, irritability). Items were considered equivalent if wording or meaning overlapped (e.g., “I felt that life was meaningless” vs. “I feel that my life has meaning”). Items capturing multiple domains (e.g., depressed mood + anhedonia) were coded under each. Discrepancies were resolved by a third expert (AC) (see supplementary).

#### 2.3.2 Scale Standardisation

Response scales varied across instruments: the CES‐D used binary responses (0 = No, 1 = Much of the time), whereas the DASS‐21 used a 4‐point Likert format (0–3). To ensure comparability, all items were binarised to the lowest common response level. CES‐D items were retained as binary, and DASS‐21 items dichotomised using a predefined threshold (Supporting Information S1: Table 1).

#### 2.3.3 Item Matching

Each construct required an equal number of conceptually comparable items per cohort. We used the Harmony online harmonisation tool (McElroy, Moltrecht et al. 2022; McElroy, Wood et al. 2024) to assess semantic similarity between items across ELSA‐UK and ELSA‐Brasil. Harmony uses the Sentence Bidirectional Encoder Representations from Transformers model (SBERT) model (Reimers and Gurevych 2019), a deep learning model optimised for natural language inference, to calculate cosine similarity which measures the cosine of the angle between their embedding vectors in multidimensional space, with values closer to 1 indicating that the items are more semantically alike, and values closer to 0 indicating little semantic overlap.

Rather than imposing a strict cut‐off, Harmony scores guided expert review. Items were judged for conceptual equivalence against DSM‐5 domains and context (e.g., scale purpose, response format). Lower‐scoring but conceptually aligned pairs (e.g., feeling apathy vs. feeling lack of motivation) were retained; higher‐scoring but nonequivalent pairs (e.g., “not feeling much worth as a person” vs. “not feeling one's life is worthwhile”) were excluded. Where multiple candidates existed, the pair best satisfying both semantic and clinical criteria was selected.

#### 2.3.4 Measurement Invariance Testing

After selecting harmonised items, we tested whether the depression and anxiety constructs were measured equivalently across cohorts and over time using multi‐group confirmatory factor analysis (MGCFA). Measurement invariance evaluates whether the relationships between latent constructs (e.g., depression) and their observed indicators are comparable (i) longitudinally within each cohort and (ii) cross‐sectionally between cohorts. Without establishing invariance, any observed differences between groups may reflect measurement artefacts rather than true differences in the latent construct (Van De Schoot et al. 2015; Putnick and Bornstein 2016).

MGCFA tests whether a construct is measured equivalently across groups by fitting progressively constrained models (Sass and Schmitt 2013). It begins with configural invariance, which assesses whether groups share the same factor structure, followed by metric invariance, where factor loadings are constrained to equality to determine whether associations with the latent construct are comparable. Scalar invariance then adds equality constraints on item thresholds or intercepts, allowing valid comparison of latent means, while strict invariance additionally constrains residual variances, though this level is rarely required (Yoon and Kim 2014). When full invariance is not achieved, partial measurement invariance can be applied by freeing selected parameters while keeping others fixed, provided at least two invariant items anchor each factor (Van De Schoot et al. 2015).

#### 2.3.5 Harmonised Measure Extraction

Once partial or full measurement invariance was established, harmonised latent factor scores were derived as cross‐cohort, longitudinally comparable indicators of depression and anxiety. Factor scores (mean = 0, SD = 1) estimate individual standing on latent constructs; higher values indicate greater symptom burden. Constraining parameters across groups and waves places scores on a shared metric, enabling cross‐cohort and temporal comparisons (Lai and Tse 2024).

### 2.4 Statistical Analyses

Longitudinal invariance was tested within each cohort: ELSA‐Brasil (COVID waves 1–4) and ELSA‐UK (waves 9, COVID 1, COVID 2, and wave 10).

We then tested cross‐cohort models using identical matched items. This ensured that the cross‐cohort models were informed by the longitudinal invariance results. Cross‐cohort models were estimated for each wave separately rather than collapsing or averaging across time points, allowing us to examine invariance within specific temporal contexts.

Model fit was evaluated using standard CFA fit indices: RMSEA, CFI, TLI, and SRMR (Hu and Bentler 1998). Following Hu and Bentler's two‐index approach, acceptable fit was defined as RMSEA ≤ 0.06; SRMR ≤ 0.08; CFI/TLI ≥ 0.95 with values of CFI/TLI > 0.90 considered adequate (Schreiber et al. 2006). Measurement invariance was evaluated using both absolute model fit and changes in fit indices between increasingly constrained models (configural, metric, and scalar). Following commonly recommended guidelines, changes in fit indices between nested models were evaluated using ΔCFI ≤ 0.010 and ΔRMSEA ≤ 0.015, and ΔSRMR ≤ 0.030 for metric invariance and ≤ 0.010 for scalar invariance (Chen 2007; G. W. Cheung and Rensvold 2002). Because recommended thresholds vary, particularly for categorical indicators estimated using the WLSMV estimator (Svetina et al. 2020), conclusions regarding invariance were based on absolute model fit, changes in fit indices, and substantive model interpretability, rather than any single criterion.

All models were estimated in *lavaan* (Rosseel 2012) using the WLSMV estimator, which is suitable for binary indicators (Kiliç et al. 2020). Analysis scripts are available at: https://github.com/cgmhc/cgmhc_harmonisation.

## 3 Results

Table 1 presents baseline descriptive statistics for each cohort. The mean age was 69 years in ELSA‐UK and 61 years in ELSA‐Brasil. Educational attainment was higher in ELSA‐Brasil, with 95% of participants educated at high school level or above, compared with 70% in ELSA‐UK.

**TABLE 1:** Descriptive statistics of demographics across ELSA‐UK and ELSA‐Brasil cohorts.

Cohort	ELSA‐UK (*N* = 7361)	ELSA‐Brasil (*N* = 2636)
Baseline year	2020	2020
Age (years, SD)	69 (10)	61.3 (8.5)
Female sex, *n* (%)	4152 (56%)	1507 (57.2%)
Education, *n* (%)	Below high school = 984 (13%) High school or higher = 5110 (70%) Missing = 1267 (17%)	Below high school = 68 (2.6%) High school or higher = 2498 (94.8%) Missing = 70 (2.6%)
Ethnicity (ethnic minority), *n* (%)	348 (5%)	853 (32.8%)
Employment (retired), *n* (%)	4660 (63%)	489 (24.1%)
Household (living alone), *n* (%)	1620 (22%)	329 (16.8%)

Table 2 summarises the Harmony item‐matching process. Five matching item pairs were identified for depression (depressed mood, anhedonia, worthlessness, hopelessness, apathy) and four for anxiety (worry, general anxiety, irritability, restlessness). Cosine similarity (Hcos) scores varied across matches, with final selections based on both semantic similarity and conceptual alignment with DSM‐5 symptom domains. Cut‐offs used to standardise and recode response categories across cohorts are shown in Table 1.

**TABLE 2:** Items matched across cohorts by semantic similarity using harmony tool.

Symptoms	ELSA‐Brasil	ELSA‐UK	Match
**Depressed mood**	**13. I Felt down‐hearted and blue (Senti‐me depressivo (a) e sem ânimo)** **^a^**	**1. Much of the time during the past week: You felt depressed** **^b^**	**0.63**
**Anhedonia**	**16. I Was unable to** **become enthusiastic about anything (Não consegui me entusiasmar com nada)** **^a^**	**2. On a scale of 0–10, where 0 is “not at all” and 10 is “very”, how satisfied are you with your life nowadays?** **^c^**	**0.69**
**Feelings of worthlessness/excessive guilt**	**17. I Felt I wasn't worth much as a person (Senti que não tinha valour como pessoa)** **^a^**	**To what extent do you feel the things you do in your life are worthwhile?** **^c^**	**0.52**
Recurrent thoughts of death	NA	NA	
**Hopelessness**	**21. I Felt that life was meaningless (Senti que a vida não tinha sentido)** **^a^**	**2. How often do you feel** **like: I feel that my life has meaning** **^d^**	**−0.73**
Fatigue or loss of energy	NA	2. Much of the time during the past week: You felt that everything you did was an effort^b^	
Insomnia or hypersomnia	NA	3. Much of the time during the past week: Your sleep was restless^b^	
Significant weight loss or weight gain	NA	NA	
**Lack of motivation or apathy**	**5. I Found it difficult to work up the initiative to do things (Achei difícil ter iniciativa para fazer as coisas)** **^a^**	**7.** **Much of the time during the past week: You could not get going** **^b^**	**0.30**
Psychomotor retardation or agitation	11. I Found myself getting agitated (Senti‐me agitado)^a^	NA	
Cognitive impairment—Diminished ability to think or concentrate	NA	NA	
Loneliness	NA	5. Much of the time during the past week: You felt lonely^b^	
Tense/stressed	Available	NA	
**Worry**	**9. I** **Was worried about situations in which I might panic and make a fool of myself (Preocupei‐me com situações em que eu pudesse entrar em pânico e parecesse ridículo)** **^a^**	**3.** **Over the last 2 weeks: Worrying too much about different things** **^e^**	**0.76**
**General anxiety**	**20. I** **Felt scared without any good reason (Senti medo sem motivo)** **^a^**	**1.** **Over the last 2 weeks: Feeling nervous, anxious or on edge** **^e^**	**0.62**
**Irritability**	**14. I Was intolerant of anything that kept me from getting on with what I was doing (Fui intolerante com as coisas que me impediam de continuar o que eu estava fazendo)** **^a^**	**6.** **Over the last 2 weeks: Becoming easily annoyed or irritable** **^e^**	**0.58**
Panic	15. I Felt I was close to panic (Senti que ia entrar em pânico)^a^	NA	
Health anxiety/perceived poor health	NA	NA	
**Restlessness**	**1. I Found it hard to wind down (Achei difícil me acalmar)** **^a^**	**4.** **Over the last 2 weeks: Trouble relaxing** **^e^**	**0.78**
Somatic complaints	2. I Was aware of dryness of my mouth (Senti minha boca seca)^a^	NA	
Situational anxiety	NA	NA	
Overwhelmed	NA	NA	
Breakdown	NA	NA	

*Note:* Bolding indicates successfully matched items that were selected for analysis.

^a^ DASS‐21.

^b^ CES‐D.

^c^ ONS‐4.

^d^ CASP‐19.

^e^ GAD‐7.

Table 3 shows longitudinal measurement invariance results within each cohort. In ELSA‐Brasil, configural, metric, and scalar models for both depression and anxiety demonstrated excellent fit (RMSEA < 0.06, CFI/TLI > 0.95). The largest observed changes in fit indices across nested models were small (ΔCFI = 0.001, ΔRMSEA = 0.002, ΔSRMR = 0.002), remaining within commonly recommended thresholds and supporting full scalar invariance across the four COVID‐19 waves. Similarly, ELSA‐UK demonstrated acceptable fit for depression and excellent fit for anxiety. The largest deterioration in fit indices (ΔCFI = 0.007, ΔRMSEA = 0.005, ΔSRMR = 0.009) was also consistent with recommended criteria for longitudinal scalar invariance.

**TABLE 3:** Fit indices for longitudinal measurement invariance of depression and anxiety within ELSA‐Brasil (*N* = 2636) and ELSA‐UK (*N* = 13,695) cohorts.

Construct	Model	χ^2^ (DF)	*p*‐value	RMSEA	CFI	TLI	SRMR
ELSA‐Brasil							
	Configural	540.061 (134)	< 0.001	0.034	0.989	0.985	0.064
Depression	Metric	562.546 (146)	< 0.001	0.033	0.989	0.986	0.066
	Scalar	608.118 (161)	< 0.001	0.032	0.988	0.986	0.066
	Configural	118.497 (39)	< 0.001	0.028	0.991	0.985	0.049
Anxiety	Metric	125.579 (45)	< 0.001	0.026	0.991	0.987	0.051
	Scalar	136.644 (53)	< 0.001	0.025	0.991	0.988	0.051
ELSA‐UK							
	Configural	1387 (134)	< 0.001	0.030	0.987	0.981	0.058
Depression	Metric	1749 (146)	< 0.001	0.032	0.983	0.978	0.067
	Scalar	2447 (161)	< 0.001	0.037	0.976	0.972	0.067
	Configural	40 (39)	0.434	0.002	1.000	1.000	0.018
Anxiety	Metric	52 (45)	0.229	0.004	1.000	1.000	0.019
	Scalar	61 (53)	0.205	0.004	1.000	1.000	0.020

Table 4 summarises cross‐cohort invariance testing. For depression, acceptable fit was maintained from configural through scalar models. The largest changes in fit indices (ΔCFI = 0.006, ΔRMSEA = 0.004, ΔSRMR = 0.010) remained within or close to commonly recommended thresholds and, together with the overall pattern of good absolute model fit, supported scalar invariance and latent mean comparisons between ELSA‐UK and ELSA‐Brasil. For anxiety, configural and metric models demonstrated excellent fit; however, the scalar model failed to converge, indicating that latent mean comparisons across cohorts could not be supported.

**TABLE 4:** Fit indices for cross‐cohort measurement invariance models of depression and anxiety in ELSA‐Brasil (*n* = 2636) and ELSA UK cohorts (*N* = 13,695).

Construct	Model	χ^2^ (DF)	*p*‐value	RMSEA	CFI	TLI	SRMR
Depression	Configural	1926.638 (268)	< 0.001	0.031	0.988	0.982	0.060
	Metric	2676.207 (296)	< 0.001	0.035	0.982	0.977	0.070
	Scalar	3219.805 (307)	< 0.001	0.038	0.978	0.973	0.068
Anxiety	Configural	158.297 (78)	< 0.001	0.013	0.999	0.998	0.025
	Metric	212.696 (93)	< 0.001	0.015	0.999	0.998	0.028
	Scalar	—	—	—	—	—	—

Table 5 shows the mean factor scores for depression across waves for ELSA‐Brasil and ELSA‐UK. Minor changes between waves are observed for both cohorts. Notably, depression factor scores for ELSA‐Brasil are substantially greater than scores for ELSA‐UK. In comparison, mean differences in raw depression scores between cohorts are much smaller.

**TABLE 5:** Mean and standard deviation of depression factor scores and raw scores for ELSA‐Brasil and ELSA‐UK across waves.

	Wave 1	Wave 2	Wave 3	Wave 4
Mean factor scores (SD)				
ELSA‐Brasil	1.067 (0.541)	1.128 (0.453)	1.071 (0.521)	1.173 (0.478)
ELSA‐UK	0.157 (0.786)	0.217 (0.853)	0.250 (0.927)	0.182 (0.853)
Mean raw scores (SD)				
ELSA‐Brasil	0.514 (1.12)	0.416 (0.99)	0.390 (0.98)	0.665 (1.23)
ELSA‐UK	0.392 (0.88)	0.463 (0.97)	0.499 (1.03)	0.364 (0.87)

**FIGURE 3:**
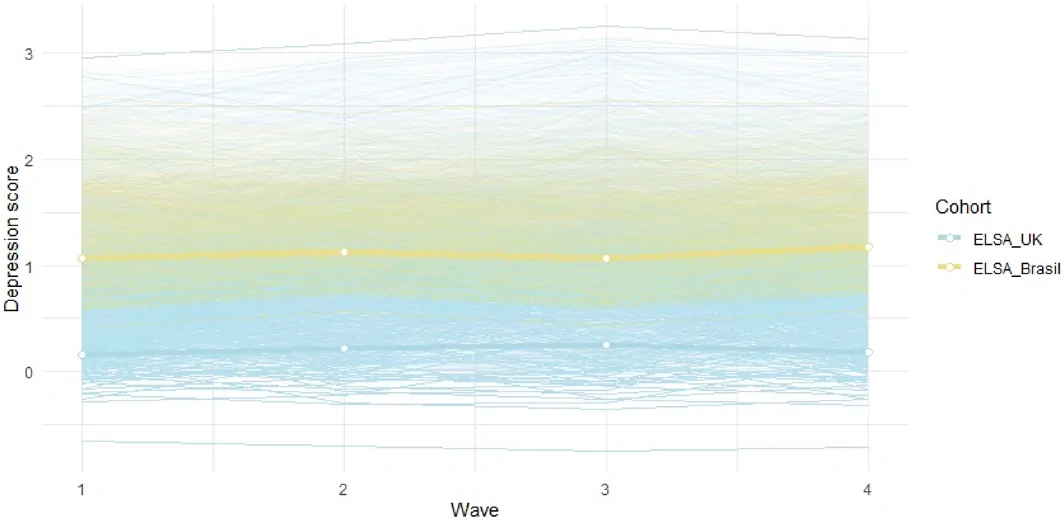
Trajectories of depression (represented by factor scores) across COVID waves for ELSA‐UK and ELSA‐Brasil cohorts.

Figure 3 shows the harmonised depression trajectories for each participant across cohorts.

## 4 Discussion

Our study illustrates the feasibility of retrospectively harmonising psychological symptom measures across culturally distinct ageing cohorts using a structured, theory‐driven approach. We combined expert item mapping with AI‐enabled semantic matching and MGCFA with the aim to harmonise cohorts with substantial differences in item content, response scales, and cultural context. In summary, we successfully derived harmonised depression, but not anxiety constructs across ELSA‐UK and ELSA‐Brasil. Depression achieved longitudinal and cross‐cohort scalar invariance, supporting comparable latent mean estimation across time and countries despite different instruments. Although some changes in fit indices approached recommended thresholds, the overall pattern of evidence supported scalar invariance for depression.

These findings extend prior harmonisation work. For example, the CLOSER collaboration successfully applied factor‐analytic harmonisation to psychological distress measures across six British cohorts (McElroy, Villadsen et al. 2020). Our results build on this by pushing beyond a single‐country context to show that a more stringent, generalisable framework can work across diverse populations. Similarly, studies in other domains have highlighted the value of advanced statistical harmonisation for cross‐national comparisons. For instance (Vonk et al. 2022), harmonised cognitive measures between the US and India, finding that linking items via CFA enabled valid cross‐country comparisons of cognitive function. In the same spirit, a depressive symptom scale (EURO‐D) achieved approximate scalar invariance across 27 European countries, supporting its use for cross‐cultural comparisons (Fong and Chan 2025). Together, these examples underscore that harmonisation methods can facilitate joint analysis of cohorts in different nations, allowing researchers to disentangle true population differences from measurement bias.

A key step in our approach was the use of an AI‐based semantic matching tool (Harmony) to aid item harmonisation. Prior work has shown that such NLP‐based tools can greatly reduce the manual effort in harmonisation: they quickly calculate high‐potential item matches, which experts can then verify (McElroy, Moltrecht et al. 2022). In our case, Harmony helped identify candidate matches across English and Portuguese scales, streamlining the mapping process. Nevertheless, we emphasise that semantic similarity does not guarantee conceptual equivalence. For example, Harmony gave a moderate similarity score (cosine ≈ 0.52) to the pairs “not feeling worth much as a person” and “not feeling one's life is worthwhile.” Although both involve the notion of worth, an expert might note that the former taps self‐esteem or self‐concept, whereas the latter reflects a sense of purpose or meaning (Ammerman et al. 2021). Such nuances are not distinguished by the embedding algorithm. Therefore, we adopted a hybrid strategy: automated matching to flag likely pairs, followed by clinician review to adjudicate subtle conceptual differences. This balance allowed us to gain efficiency from the algorithm while preserving domain fidelity. Harmony also depends on the quality of translated item wording and metadata; semantic similarity should therefore complement, rather than replace, expert judgement.

We recognise several limitations with our approach. Although we achieved configural and metric invariance for anxiety, our cross‐cohort scalar model for anxiety did not converge. This is consistent with previous cross‐cultural measurement invariance studies showing that full scalar invariance is often difficult to achieve across culturally diverse populations, even when identical instruments are administered, likely reflecting genuine differences in symptom interpretation, response styles, and cultural conceptualisations of mental health (Murray et al. 2022; Katus et al. 2022). This result may also reflect the smaller number and greater heterogeneity of overlapping anxiety items available between ELSA‐UK and ELSA‐Brasil. Insufficient item coverage can preclude full invariance: with only a handful of anxiety items, the model lacked power to align thresholds across groups. This failure highlights a general point: successful harmonisation requires a reasonably large pool of well‐matched items. When instruments are too dissimilar or sparse, one may only achieve partial invariance. In practice, partial invariance can sometimes be acceptable; with guidelines suggesting that having at least half of the indicators invariant may allow for meaningful comparisons (Little 2024). In extreme cases, problematic items might need to be dropped or given less weight in scoring (Zhao et al. 2023). Ultimately, our anxiety results illustrate the difficulty of retrospectively harmonising constructs that were measured inconsistently across studies.

Furthermore, it is important to acknowledge the assumptions and trade‐offs in our approach. Our framework is anchored in a priori decisions about which items captured the same symptom domains in both countries. These judgments assumed that, despite different wordings and answer formats, each paired item tapped an equivalent underlying construct. Although validated Brazilian Portuguese versions of the DASS‐21 were used in ELSA‐Brasil, conceptual differences arising from language and cultural interpretation may still contribute to measurement variation beyond differences between instruments. We also collapsed response categories into binary indicators to allow direct pooling; this simplification undoubtedly sacrificed some information in exchange for comparability (Purgato and Barbui 2013). Thus, the validity of our harmonised scores depends critically on the soundness of these initial decisions. Future users of this framework should be transparent about such choices and consider sensitivity analyses (e.g., testing alternative coding schemes) when feasible.

Alternative harmonisation strategies may also address some of the limitations of our approach, although each involves different assumptions and trade‐offs. For example, integrative data analysis (IDA) pools raw data across studies (Curran and Hussong 2009), while test equating and linking methods align scales using anchor items or external calibration samples (Kolen and Brennan 2014). Meta‐analytic approaches can synthesise study‐level results when individual‐level data are unavailable but do not support longitudinal modelling or formal measurement invariance testing (M.‐W.L. Cheung and Cheung 2016). Multiple‐group item response theory (IRT) provides a more granular alternative by evaluating differential item functioning, but requires stronger unidimensionality assumptions and larger sample sizes (Muthén and Asparouhov 2014; Kim and Yoon 2011).

In summary, our structured harmonisation framework proved efficient and robust for depression: despite different instruments and languages, we derived a common latent depression metric that behaves consistently. For our anxiety construct, we were able to detect that invariance was not met, thus avoiding forcibly harmonising conceptually inconsistent data. However, the failure to achieve cross‐cohort scalar invariance for anxiety underscores the limits of retrospective harmonisation, especially when instruments diverge. These issues would only multiply as more cohorts or countries enter the analysis, reducing the chance of finding matching items across all datasets. Therefore, future studies would benefit from prospective harmonisation efforts: agreeing on core symptom domains and standardised item sets (e.g., using DSM‐based criteria or well‐validated global mental health modules) at the design phase. Adopting common measurement frameworks or shared modules across international studies would minimise data loss and enhance comparability from the outset. Until then, our results show that careful, methodical harmonisation, combining expert knowledge, NLP tools, and factor analysis, can nevertheless yield meaningful cross‐national mental health metrics.

## Author Contributions

**James Lian:** conceptualization, methodology, formal analysis, investigation, validation, visualization, writing – original draft, writing – review and editing. **Pedro F. Zuccolo:** conceptualization, methodology, validation, investigation, formal analysis, visualization, writing – original draft, writing – review and editing. **Omid V. Ebrahimi:** conceptualization, methodology, supervision, writing – review and editing, investigation. **Daniel Fatori:** conceptualization, methodology, writing – review and editing, investigation. **Abhaya Adlakha:** data curation, methodology. **Juan F. De La Hoz:** conceptualization, methodology, investigation, writing – review and editing. **Younga H. Lee:** conceptualization, methodology, investigation, supervision, project administration, writing – review and editing. **Adriana Carneiro:** funding acquisition. **Isabela M. Benseñor:** funding acquisition. **Paulo A. Lotufo:** funding acquisition. **Alessandra C. Goulart:** funding acquisition. **Justin D. Tubbs:** writing – review and editing. **Devon Watts:** writing – review and editing. **Yu Zhou:** writing – review and editing. **Lorenza Dall’Aglio:** writing – review and editing. **Mihael Cudic:** writing – review and editing. **Morgane Kuenzi:** writing – review and editing. **Ronald C. Kessler:** supervision, methodology, writing – review and editing. **Vikram Patel:** supervision, methodology, writing – review and editing. **André Brunoni:** supervision, methodology, funding acquisition, conceptualization. **Jordan W. Smoller:** funding acquisition, writing – review and editing, supervision, project administration, conceptualization. **Sarah Bauermeister:** funding acquisition, supervision, conceptualization.

## Funding

This study was funded by the National Institute of Mental Health—1RF1MH134638. PFZ has received funds from Fundação de Amparo à Pesquisa do Estado de São Paulo (FAPESP, Grant No. 2024/17532‐0). DF has received funds from Wellcome Leap 1kD. ELSA‐Brasil was funded by National Council for Scientific and Technological Development (CNPq) (Wave 1: BA 01 06 021200; ES 01 06 0300‐00; MG 01 06 0278‐00; 01 06 0071‐00; RS 01 06 0010‐00; SP 01 06 0115‐00; FAPESP 2020/01476‐2).

## Ethics Statement

The authors assert that all procedures contributing to this work comply with the ethical standards of the relevant national and institutional committees on human experimentation and with the Helsinki Declaration of 1975, as revised in 200.

## Conflicts of Interest

The authors declare no conflicts of interest.

## Supporting information


Supporting Information S1

## Data Availability

Data from ELSA‐Brasil and ELSA‐UK can be accessed via application to DPUK. See: https://portal.dementiasplatform.uk/apply‐for‐access/.

## References

[mpr70102-bib-0001] Alavi, M. , D. Le Lagadec , and M. Cleary . 2026. “Challenges of Cross‐Cultural Validation of Clinical Assessment Measures: A Practical Introduction.” Journal of Advanced Nursing 82, no. 1: 941–949. 10.1111/jan.16906. 40129093 PMC12721952

[mpr70102-bib-0002] Almeida, M. , A. D. Shrestha , D. Stojanac , and L. J. Miller . 2020. “The Impact of the COVID‐19 Pandemic on Women’s Mental Health.” Archives of Women's Mental Health 23, no. 6: 741–748. 10.1007/s00737-020-01092-2. PMC7707813 33263142

[mpr70102-bib-0003] Ammerman, B. A. , T. A. Burke , R. Jacobucci , and K. McClure . 2021. “How we Ask Matters: The Impact of Question Wording in Single‐Item Measurement of Suicidal Thoughts and Behaviors.” Preventive Medicine 152, no. Pt 1: 106472. 10.1016/j.ypmed.2021.106472. 34538365

[mpr70102-bib-0004] Batterham, P. J. , A. L. Calear , S. M. McCallum , et al. 2021. “Trajectories of Depression and Anxiety Symptoms During the COVID‐19 Pandemic in a Representative Australian Adult Cohort.” Medical Journal of Australia 214 no. 10: 462–468. https://onlinelibrary.wiley.com/doi/10.5694/mja2.51043. 33899939 10.5694/mja2.51043 PMC8207103

[mpr70102-bib-0005] Bauermeister, S. , M. Phatak , K. Sparks , et al. 2023. “Evaluating the Harmonisation Potential of Diverse Cohort Datasets.” European Journal of Epidemiology 38, no. 6: 605–615. 10.1007/s10654-023-00997-3. 37099244 PMC10232583

[mpr70102-bib-0006] Black, M. H. , J. Buitelaar , T. Charman , et al. 2024. “Conceptual Framework for Data Harmonisation in Mental Health Using the International Classification of Functioning, Disability and Health: An Example with the R2D2‐MH Consortium.” BMJ mental health 27, no. 1: e301283. 10.1136/bmjment-2024-301283. PMC11603809 39608798

[mpr70102-bib-0007] Brunoni, A. R. , P. J. C. Suen , P. S. Bacchi , et al. 2023. “Prevalence and Risk Factors of Psychiatric Symptoms and Diagnoses Before and During the COVID‐19 Pandemic: Findings From the ELSA‐Brasil COVID‐19 Mental Health Cohort.” Psychological Medicine 53, no. 2: 446–457. 10.1017/S0033291721001719. 33880984 PMC8144814

[mpr70102-bib-0008] Bu, F. , A. Steptoe , and D. Fancourt . 2023. “Depressive and Anxiety Symptoms in Adults During the COVID‐19 Pandemic in England: A Panel Data Analysis Over 2 Years.” PLoS Medicine 20, no. 4: e1004144. 10.1371/journal.pmed.1004144. 37071605 PMC10112796

[mpr70102-bib-0009] Cénat, J. M. , S. M. M. M. Farahi , R. D. Dalexis , et al. 2022. “The Global Evolution of Mental Health Problems During the COVID‐19 Pandemic: A Systematic Review and Meta‐Analysis of Longitudinal Studies.” Journal of Affective Disorders 315: 70–95. 10.1016/j.jad.2022.07.011. 35842064 PMC9278995

[mpr70102-bib-0010] Chen, F. F. 2007. “Sensitivity of Goodness of Fit Indexes to Lack of Measurement Invariance.” Structural Equation Modeling: A Multidisciplinary Journal 14, no. 3: 464–504. 10.1080/10705510701301834.

[mpr70102-bib-0011] Cheng, C. , L. Messerschmidt , I. Bravo , et al. 2024. “A General Primer for Data Harmonization.” Scientific Data 11, no. 1: 152. 10.1038/s41597-024-02956-3. 38297013 PMC10831085

[mpr70102-bib-0012] Cheung, G. W. , and R. B. Rensvold . 2002. “Evaluating Goodness‐of‐fit Indexes for Testing Measurement Invariance.” Structural Equation Modeling 9, no. 2: 233–255. 10.1207/s15328007sem0902_5.

[mpr70102-bib-0013] Cheung, M. W.‐L. , and S. F. Cheung . 2016. “Random‐Effects Models for Meta‐Analytic Structural Equation Modeling: Review, Issues, and Illustrations.” Research Synthesis Methods 7, no. 2: 140–155. 10.1002/jrsm.1166. 27286900

[mpr70102-bib-0014] Curran, P. J. , and A. M. Hussong . 2009. “Integrative Data Analysis: The Simultaneous Analysis of Multiple Data Sets.” Psychological Methods 14, no. 2: 81–100. 10.1037/a0015914. 19485623 PMC2777640

[mpr70102-bib-0015] Davidov, E. , B. Meuleman , J. Cieciuch , P. Schmidt , and J. Billiet . 2014. “Measurement Equivalence in Cross‐National Research.” Annual Review of Sociology 40, no. 1: 55–75. 10.1146/annurev-soc-071913-043137.

[mpr70102-bib-0016] Dementias Platform UK . 2024. https://www.dementiasplatform.uk/about‐us/about‐us.

[mpr70102-bib-0017] Ebrahimi, O. V. , D. J. Bauer , A. Hoffart , and S. U. Johnson . 2022. “A Critical Period for Pandemic Adaptation: The Evolution of Depressive Symptomatology in a Representative Sample of Adults Across a 17‐month Period During COVID‐19.” Journal of Psychopathology and Clinical Science 131, no. 8: 881–894. 10.1037/abn0000786. 36326629

[mpr70102-bib-0018] Fancourt, D. , A. Steptoe , and F. Bu . 2021. “Trajectories of Anxiety and Depressive Symptoms During Enforced Isolation due to COVID‐19 in England: A Longitudinal Observational Study.” Lancet Psychiatry 8, no. 2: 141–149. 10.1016/S2215-0366(20)30482-X. 33308420 PMC7820109

[mpr70102-bib-0019] Fatori, D. , P. Suen , P. Bacchi , et al. 2022. “Trajectories of Common Mental Disorders Symptoms Before and During the COVID‐19 Pandemic: Findings From the ELSA‐Brasil COVID‐19 Mental Health Cohort.” Social Psychiatry and Psychiatric Epidemiology 57, no. 12: 2445–2455. 10.1007/s00127-022-02365-0. 36114857 PMC9483303

[mpr70102-bib-0020] Fong, T. C. T. , and R. T. H. Chan . 2025. “Longitudinal Measurement Invariance of EURO‐D Scale Across 27 Countries in SHARE Wave 8 and Wave 9: A Cross‐Country Alignment Study.” Journal of Affective Disorders 390, no. 119852: 119852. 10.1016/j.jad.2025.119852. 40639546

[mpr70102-bib-0021] Fortier, I. , D. Doiron , P. Burton , and P. Raina . 2011. “Invited Commentary: Consolidating Data harmonization—How to Obtain Quality and Applicability?” American Journal of Epidemiology 174, no. 3: 261–264. 10.1093/aje/kwr194. 21749975

[mpr70102-bib-0022] Fortier, I. , P. Raina , E. R. Van den Heuvel , et al. 2017. “Maelstrom Research Guidelines for Rigorous Retrospective Data Harmonization.” International Journal of Epidemiology 46, no. 1: 103–105. https://pubmed.ncbi.nlm.nih.gov/27272186/. 27272186 10.1093/ije/dyw075 PMC5407152

[mpr70102-bib-0023] Hale, T. , N. Angrist , B. Kira , A. Petherick , T. Phillips , and S. Webster . 2020. “Variation in Government Responses to COVID‐19.” https://www.bsg.ox.ac.uk/sites/default/files/2020‐12/BSG‐WP‐2020‐032‐v10.pdf. 10.1371/journal.pone.0253116 PMC8270409 34242239

[mpr70102-bib-0025] Hawks, J. L. 2023. “Editorial: The Impact of the COVID‐19 Pandemic on Racial Disparities in Pediatric Mental Health.” Journal of the American Academy of Child & Adolescent Psychiatry 62, no. 4: 398–399. 10.1016/j.jaac.2022.12.015. 36608739 PMC9807287

[mpr70102-bib-0026] Hawrilenko, M. , E. Kroshus , P. Tandon , and D. Christakis . 2021. “The Association Between School Closures and Child Mental Health During COVID‐19.” JAMA Network Open 4, no. 9: e2124092. 10.1001/jamanetworkopen.2021.24092. 34477850 PMC8417763

[mpr70102-bib-0027] Hu, L.‐T. , and P. M. Bentler . 1998. “Fit Indices in Covariance Structure Modeling: Sensitivity to Underparameterized Model Misspecification.” Psychological Methods 3, no. 4: 424–453. 10.1037/1082-989x.3.4.424.

[mpr70102-bib-0028] Iob, E. , P. Frank , A. Steptoe , and D. Fancourt . 2020. “Levels of Severity of Depressive Symptoms Among At‐Risk Groups in the UK During the COVID‐19 Pandemic.” JAMA Network Open 3, no. 10: e2026064. 10.1001/jamanetworkopen.2020.26064. 33104209 PMC7588938

[mpr70102-bib-0076] Katus, L. , S. Foley , A. L. Murray , et al. 2022. “Perceived Stress During the Prenatal Period: Assessing Measurement Invariance of the Perceived Stress Scale (PSS‐10) Across Cultures and Birth Parity: Katus et al.” Archives of Women's Mental Health 25, no. 3: 633–640. 10.1007/s00737-022-01229-5 PMC9072510 35420323

[mpr70102-bib-0029] Kennedy, E. , S. Vadlamani , H. M. Lindsey , et al. 2024. “Bridging Big Data in the ENIGMA Consortium to Combine Non‐Equivalent Cognitive Measures.” Scientific Reports 14, no. 1: 24289. 10.1038/s41598-024-72968-x. 39414844 PMC11484938

[mpr70102-bib-0030] Kiliç, A. , İ. Uysal , and B. Atar . 2020. “Comparison of Confirmatory Factor Analysis Estimation Methods on Binary Data.” International journal of assessment tools in education 7, no. 3: 451–487. 10.21449/ijate.660353.

[mpr70102-bib-0031] Kim, E. S. , and M. Yoon . 2011. “Testing Measurement Invariance: A Comparison of Multiple‐Group Categorical CFA and IRT.” Structural Equation Modeling: A Multidisciplinary Journal 18, no. 2: 212–228. 10.1080/10705511.2011.557337.

[mpr70102-bib-0032] King, K. , N. Allum , P. Stoneman , and A. Cernat . 2023. “Estimating Measurement Equivalence of the 12‐Item General Health Questionnaire Across Ethnic Groups in the UK.” Psychological Medicine 53, no. 5: 1778–1786. 10.1017/S0033291721003408. 34498557 PMC10106293

[mpr70102-bib-0033] Kolen, M. J. , and R. L. Brennan . 2014. Test Equating, Scaling, and Linking: Methods and Practices 3rd Ed. Springer. 10.1007/978-1-4939-0317-7.

[mpr70102-bib-0034] Lai, M. H. C. , and W. W.‐Y. Tse . 2024. “Are Factor Scores Measurement Invariant?” Psychological Methods. 10.1037/met0000658. 38709627

[mpr70102-bib-0035] Lee, J. , J. Wilkens , D. Phillips , D. Knapp , and E. Nichols . 2024. “Recommended Contents for Maximizing Harmonization Potential for the Health and Retirement Study and Its International Network of Studies.” Cesr‐Schaeffer Working Paper Series. https://papers.ssrn.com/sol3/papers.cfm?abstract_id=4986755.

[mpr70102-bib-0036] Lee, Y. H. , Z. Liu , D. Fatori , et al. 2022. “Association of Everyday Discrimination With Depressive Symptoms and Suicidal Ideation During the COVID‐19 Pandemic in the all of Us Research Program.” JAMA Psychiatry 79, no. 9: 898–906. 10.1001/jamapsychiatry.2022.1973. 35895053 PMC9330278

[mpr70102-bib-0038] Leung, C. M. C. , M. K. Ho , A. A. Bharwani , et al. 2022. “Mental Disorders Following COVID‐19 and Other Epidemics: A Systematic Review and Meta‐Analysis.” Translational Psychiatry 12, no. 1: 205. 10.1038/s41398-022-01946-6. 35581186 PMC9110635

[mpr70102-bib-0040] Little, T. D. 2024. Longitudinal Structural Equation Modeling (Methodology in the Social Sciences). Guilford Press.

[mpr70102-bib-0041] Maelstrom Research . 2024. http://www.maelstrom‐research.org/.

[mpr70102-bib-0042] Magnúsdóttir, I. , A. Lovik , A. B. Unnarsdóttir , et al. 2022. “Acute COVID‐19 Severity and Mental Health Morbidity Trajectories in Patient Populations of Six Nations: An Observational Study.” Lancet Public Health 7, no. 5: e406–e416. 10.1016/S2468-2667(22)00042-1. 35298894 PMC8920517

[mpr70102-bib-0078] McElroy, E. , B. Moltrecht , G. B. Ploubidis , M. Scopel Hoffman , T. A. Wood . 2022, Harmony [Computer software], Version 1.0. Ulster University . https://app.harmonydata.org.

[mpr70102-bib-0043] McElroy, E. , A. Villadsen , P. Patalay , et al. 2020. “Harmonisation and Measurement Properties of Mental Health Measures in Six British Cohorts.” https://www.closer.ac.uk/wp‐content/uploads/210715‐Harmonisation‐measurement‐properties‐mental‐health‐measures‐british‐cohorts.pdf.

[mpr70102-bib-0044] McElroy, E. , T. Wood , R. Bond , et al. 2024. “Using Natural Language Processing to Facilitate the Harmonisation of Mental Health Questionnaires: A Validation Study Using Real‐World Data.” BMC Psychiatry 24, no. 1: 530. 10.1186/s12888-024-05954-2. 39049010 PMC11267737

[mpr70102-bib-0045] Miao, R. , C. Liu , J. Zhang , and H. Jin . 2023. “Impact of the COVID‐19 Pandemic on the Mental Health of Children and Adolescents: A Systematic Review and Meta‐Analysis of Longitudinal Studies.” Journal of Affective Disorders 340: 914–922. 10.1016/j.jad.2023.08.070. 37598714

[mpr70102-bib-0046] Moltrecht, B. , J. Villanova do Amaral , G. A. Salum , et al. 2024. “Social Connection and Its Prospective Association With Adolescent Internalising and Externalising Symptoms: An Exploratory Cross‐Country Study Using Retrospective Harmonisation.” Journal of Child Psychology and Psychiatry and Allied Disciplines 66, no. 5: 725–736. 10.1111/jcpp.14080. 39644141 PMC12018293

[mpr70102-bib-0075] Murray, A. L. , C. L. Hemady , H. Do , et al. 2022. “Measuring Antenatal Depressive Symptoms Across the World: A Validation and Cross‐Country Invariance Analysis of the Patient Health Questionnaire‐9 (PHQ‐9) in Eight Diverse Low‐Resource Settings.” Psychological Assessment 34, no. 11: 993–1007. 10.1037/pas0001154. 36227303

[mpr70102-bib-0047] Muthén, B. , and T. Asparouhov . 2014. “IRT Studies of Many Groups: The Alignment Method.” Frontiers in Psychology 5: 978. 10.3389/fpsyg.2014.00978. 25309470 PMC4162377

[mpr70102-bib-0048] O’Connor, M. , E. Spry , G. Patton , et al. 2022. “Better Together: Advancing Life Course Research Through Multi‐Cohort Analytic Approaches.” Advances in Life Course Research 53, no. 100499: 100499. 10.1016/j.alcr.2022.100499. 36652217

[mpr70102-bib-0049] O’Halloran, A. M. , R. A. Kenny , and B. L. King‐Kallimanis . 2014. “The Latent Factors of Depression From the Short Forms of the CES‐D Are Consistent, Reliable and Valid in Community‐Living Older Adults.” European geriatric medicine 5, no. 2: 97–102. 10.1016/j.eurger.2013.12.004.

[mpr70102-bib-0050] Personal well‐being user guidance . 2025. https://www.ons.gov.uk/peoplepopulationandcommunity/wellbeing/methodologies/personalwellbeingsurveyuserguide.

[mpr70102-bib-0051] Phiri, P. , R. Ramakrishnan , S. Rathod , et al. 2021. “An Evaluation of the Mental Health Impact of SARS‐CoV‐2 on Patients, General Public and Healthcare Professionals: A Systematic Review and Meta‐Analysis.” EClinicalMedicine 34: 100806. 10.1016/j.eclinm.2021.100806. 33842872 PMC8022621

[mpr70102-bib-0052] Pierce, M. , S. McManus , H. Hope , et al. 2021. “Mental Health Responses to the COVID‐19 Pandemic: A Latent Class Trajectory Analysis Using Longitudinal UK Data.” Lancet Psychiatry 8, no. 7: 610–619. 10.1016/S2215-0366(21)00151-6. 33965057 PMC9764381

[mpr70102-bib-0054] Purgato, M. , and C. Barbui . 2013. “Dichotomizing Rating Scale Scores in Psychiatry: A Bad Idea?” Epidemiology and Psychiatric Sciences 22, no. 1: 17–19. 10.1017/S2045796012000613. 23089232 PMC6998334

[mpr70102-bib-0055] Putnick, D. L. , and M. H. Bornstein . 2016. “Measurement Invariance Conventions and Reporting: The State of the Art and Future Directions for Psychological Research.” Developmental Review: Developmental Review 41: 71–90. 10.1016/j.dr.2016.06.004. 27942093 PMC5145197

[mpr70102-bib-0056] Reimers, N. , and I. Gurevych . 2019. “Sentence‐BERT: Sentence Embeddings Using Siamese BERT‐Networks.” arXiv [cs.CL]. http://arxiv.org/abs/1908.10084.

[mpr70102-bib-0057] Robinson, E. , A. R. Sutin , M. Daly , and A. Jones . 2022. “A Systematic Review and Meta‐Analysis of Longitudinal Cohort Studies Comparing Mental Health Before Versus During the COVID‐19 Pandemic in 2020.” Journal of Affective Disorders 296: 567–576. 10.1016/j.jad.2021.09.098. 34600966 PMC8578001

[mpr70102-bib-0077] Rosseel, Y. 2012. “lavaan: An R Package for Structural Equation Modeling.” Journal of Statistical Software 48: 1–36.

[mpr70102-bib-0058] Santomauro, D. F. , A. M. Mantilla Herrera , J. Shadid , et al. 2021. “Global Prevalence and Burden of Depressive and Anxiety Disorders in 204 Countries and Territories in 2020 due to the COVID‐19 Pandemic.” Lancet 398, no. 10312: 1700–1712. 10.1016/s0140-6736(21)02143-7. 34634250 PMC8500697

[mpr70102-bib-0059] Sass, D. A. , and T. A. Schmitt . 2013. “Testing Measurement and Structural Invariance.” In Handbook of Quantitative Methods for Educational Research, 315–345. SensePublishers.

[mpr70102-bib-0060] Schmidt, M. I. , B. B. Duncan , J. G. Mill , et al. 2015. “Cohort Profile: Longitudinal Study of Adult Health (ELSA‐Brasil).” International Journal of Epidemiology 44, no. 1: 68–75. 10.1093/ije/dyu027. 24585730 PMC4339754

[mpr70102-bib-0061] Schreiber, J. B. , A. Nora , F. K. Stage , E. A. Barlow , and J. King . 2006. “Reporting Structural Equation Modeling and Confirmatory Factor Analysis Results: A Review.” Journal of Educational Research 99, no. 6: 323–338. 10.3200/joer.99.6.323-338.

[mpr70102-bib-0062] Spitzer, R. L. , K. Kroenke , J. B. W. Williams , and B. Löwe . 2006. “A Brief Measure for Assessing Generalized Anxiety Disorder: The GAD‐7: The GAD‐7.” Archives of Internal Medicine 166, no. 10: 1092–1097. 10.1001/archinte.166.10.1092. 16717171

[mpr70102-bib-0063] Steptoe, A. , E. Breeze , J. Banks , and J. Nazroo . 2013. “Cohort Profile: The English Longitudinal Study of Ageing.” International Journal of Epidemiology 42, no. 6: 1640–1648. 10.1093/ije/dys168. 23143611 PMC3900867

[mpr70102-bib-0064] Svetina, D. , L. Rutkowski , and D. Rutkowski . 2020. “Multiple‐Group Invariance With Categorical Outcomes Using Updated Guidelines: An Illustration Using M plus and the lavaan/semtools Packages.” Structural Equation Modeling: A Multidisciplinary Journal 27, no. 1: 111–130. 10.1080/10705511.2019.1602776.

[mpr70102-bib-0065] Van De Schoot, R. , P. Schmidt , A. De Beuckelaer , K. Lek , and M. Zondervan‐Zwijnenburg . 2015. “Editorial: Measurement Invariance.” Frontiers in Psychology 6: 1064. 10.3389/fpsyg.2015.01064. 26283995 PMC4516821

[mpr70102-bib-0066] Vignola, R. C. B. , and A. M. Tucci . 2014. “Adaptation and Validation of the Depression, Anxiety and Stress Scale (DASS) to Brazilian Portuguese.” Journal of Affective Disorders 155: 104–109. 10.1016/j.jad.2013.10.031. 24238871

[mpr70102-bib-0067] Vonk, J. M. J. , A. L. Gross , A. R. Zammit , et al. 2022. “Cross‐National Harmonization of Cognitive Measures Across HRS HCAP (USA) and LASI‐DAD (India).” PLoS One 17, no. 2: e0264166. 10.1371/journal.pone.0264166. 35213581 PMC8880818

[mpr70102-bib-0068] Welzel, C. , L. Brunkert , S. Kruse , and R. F. Inglehart . 2023. “Non‐Invariance? An Overstated Problem With Misconceived Causes.” Sociological Methods & Research 52, no. 3: 1368–1400. 10.1177/0049124121995521.

[mpr70102-bib-0069] Wey, T. W. , D. Doiron , R. Wissa , et al. 2021. “Overview of Retrospective Data Harmonisation in the MINDMAP Project: Process and Results.” Journal of Epidemiology & Community Health 75, no. 5: 433–441. 10.1136/jech-2020-214259. 33184054 PMC8053335

[mpr70102-bib-0070] Wiggins, R. D. , G. Netuveli , M. Hyde , P. Higgs , and D. Blane . 2008. “The Evaluation of a Self‐Enumerated Scale of Quality of Life (CASP‐19) in the Context of Research on Ageing: A Combination of Exploratory and Confirmatory Approaches.” Social Indicators Research 89, no. 1: 61–77. 10.1007/s11205-007-9220-5.

[mpr70102-bib-0072] Yoon, M. , and E. S. Kim . 2014. “A Comparison of Sequential and Nonsequential Specification Searches in Testing Factorial Invariance.” Behavior Research Methods 46, no. 4: 1199–1206. 10.3758/s13428-013-0430-2. 24356995

[mpr70102-bib-0073] Zhao, X. , S. Coxe , M. H. Sibley , C. Zulauf‐McCurdy , and J. W. Pettit . 2023. “Harmonizing Depression Measures Across Studies: A Tutorial for Data Harmonization.” Prevention Science: the official journal of the Society for Prevention Research 24, no. 8: 1569–1580. 10.1007/s11121-022-01381-5. 35798992 PMC9823146

[mpr70102-bib-0074] Zuccolo, P. F. , C. B. Casella , D. Fatori , et al. 2022. Children and Adolescents’ Emotional Problems During the COVID‐19 Pandemic in Brazil. European child & adolescent psychiatry. https://link.springer.com/10.1007/s00787‐022‐02006‐6. 10.1007/s00787-022-02006-6 PMC9135594 35618973

